# Variation of GP antibiotic prescribing tendency for contacts with out-of-hours primary care in Denmark – a cross-sectional register-based study

**DOI:** 10.1080/02813432.2022.2073981

**Published:** 2022-06-15

**Authors:** Linda Huibers, Claus Høstrup Vestergaard, Ellen Keizer, Bodil Hammer Bech, Flemming Bro, Morten Bondo Christensen

**Affiliations:** a Research Unit for General Practice, Aarhus, Denmark; b Department of General Practice, Institute for Public Health, Aarhus University, Aarhus, Denmark; c Research Unit for Epidemiology, Department of Public Health, Aarhus University, Aarhus, Denmark

**Keywords:** Out-of-hours medical care, primary health care, physicians, prescriptions, general practice, Denmark, anti-bacterial agents, infections

## Abstract

**Objective:**

To study variation in antibiotic prescribing rates among general practitioners (GP) in out-of-hours (OOH) primary care and to explore GP characteristics associated with these rates.

**Design:**

Population-based observational registry study using routine data from the OOH primary care registration system on patient contacts and antibiotic prescriptions combined with national register data.

**Setting:**

OOH primary care of the Central Denmark Region.

**Subjects:**

All patient contacts in 2014–2017.

**Main outcome measures:**

GPs’ tendency to prescribe antibiotics. Excess variation (not attributable to chance).

**Results:**

We included 794,220 clinic consultations (16.1% with antibiotics prescription), 281,141 home visits (11.6% antibiotics), and 1,583,919 telephone consultations (5.8% antibiotics). The excess variation in the tendency to prescribe antibiotics was 1.56 for clinic consultations, 1.64 for telephone consultations, and 1.58 for home visits. Some GP characteristics were significantly correlated with a higher tendency to prescribe antibiotics, including ‘activity level’ (i.e. number of patients seen in the past hour) for clinic and telephone consultations, ‘familiarity with OOH care’ (i.e. number of OOH shifts in the past 180 days), male sex, and younger age for home visits. Overall, GP characteristics explained little of the antibiotic prescribing variation seen among GPs (Pseudo *r*^2^: 0.008–0.025).

**Conclusion:**

Some variation in the GPs’ tendency to prescribe antibiotics was found for OOH primary care contacts. Available GP characteristics, such as GPs’ activity level and familiarity with OOH care, explained only small parts of this variation. Future research should focus on identifying factors that can explain this variation, as this knowledge could be used for designing interventions.

**KEY POINTS**
- Current awareness:
- Antibiotic prescribing rates seem to be higher in out-of-hours than in daytime primary care.
- Most important results:
- Antibiotic prescribing rates varied significantly among general practitioners after adjustment for contact- and patient-characteristics.
- This variation remained even after accounting for variation attributable to chance.
- General practitioners’ activity level and familiarity with out-of-hours care were positively associated with their tendency to prescribe antibiotics.

## Introduction

High exposure to antibiotics and inappropriate antibiotic prescribing are major causes of antibiotic resistance [1]. Antibiotic resistance may delay or reduce effective treatment, resulting in higher morbidity and mortality rates, more complications, and longer hospital stays in addition to higher healthcare costs [1,2]. Therefore, increased focus is warranted on ensuring prudent use of antibiotics, including narrow-spectrum antibiotics, and avoiding unnecessary treatment.

In Denmark, most prescriptions are issued to patients in the primary care setting [3]. GPs have varying antibiotic prescribing rates [4,5]. Gjelstad et al. found a three times difference between the lowest and highest prescribers for respiratory tract infections in the daytime [6]. A range of factors was associated with GP variation in antibiotic prescribing rates in the daytime, such as GP gender and age, years of practice experience, the volume of practice, perceived time available per patient, the general attitude towards prescribing, geographical location, consultation rates for infections, and workload [7–11].

Antibiotic prescribing is a challenge to GPs in the daytime, but even more so in the out-of-hours (OOH) services [12,13]. In 2011–2012, 15% of all contacts to Danish OOH primary care ended with an antibiotic prescription [14]. The average antibiotic prescribing rate in OOH primary care varies across Europe, ranging from 15% in Norway and England to 23.7% in Belgium and 33% in Iceland [5,15–17].

Variation in prescription rates and decisive influential factors may differ between daytime and out-of-hours care. Antibiotic prescribing rates seem to be higher in OOH than in daytime care [12,13] due to contact characteristics (e.g. overrepresentation of respiratory tract infections, more vulnerable or foreign-language patients, and more young children in OOH care) and organisational factors (e.g. high workload, limited access to patient records and to diagnostics, including point-of-care (POC) tests) [12,18–20]. In the OOH setting, GPs do not always feel sufficiently confident to use the ‘wait and see’ approach [21], and many have lower thresholds for prescribing antibiotics than during office hours as such prescriptions may provide a safety net [19,22].

Knowledge about the variation in antibiotic prescribing among GPs and associated factors can inform future intervention development. Therefore, we aimed to study GP variation in antibiotic prescribing rates and explore GP characteristics associated with antibiotic prescribing in OOH primary care. We hypothesised that the antibiotic prescribing rates varied significantly among GPs in OOH primary care.

## Material and methods

### Design and population

We conducted a population-based observational registry study using routine registry data from the OOH registration system on antibiotic prescriptions for patient contacts with the OOH primary care service (GP cooperative) in the Central Denmark Region from 1 January 2014 to 31 December 2017.

### Setting

OOH primary care provides primary healthcare outside the GPs’ office hours for all citizens in the region (1.3 million citizens on 1 January 2014) [23]. At the OOH primary care service, GPs answer the telephone and perform triage. This GP can decide to give telephone advice (telephone consultation) or refer to a face-to-face consultation with a GP (clinic consultation or home visit). The OOH primary care service is open on weekdays between 4pm and 8am, on weekends, and on holidays.

The above model exists in four out of five Danish regions. In 2014, the Capital Region of Denmark implemented a different model called medical helpline 1813, where nurses with computerised decision support and physicians answer the telephone and perform triage. Patients referred to a clinic consultation are seen at the hospital.

In Denmark, primary care is tax-funded and freely available for residents. General practice is responsible for providing healthcare to all patients 24/7. In the Central Denmark Region, all GPs (excluding GPs aged 60+ years) are assigned at least six shifts at the OOH primary care service over a 4-month period. Furthermore, GPs are allowed to swap or sell shifts.

### Data collection

We retrieved data on contacts and antibiotic prescribing from the OOH primary care registration system in the Central Denmark Region. For each contact, we included the date and time of the contact, contact type, patients’ age, and sex, GPs’ age and sex, GPs authorisation ID, and antibiotic prescription (Anatomical Therapeutic Chemical (ATC) code).

From Danish national registries, we collected additional data, which were used as covariates. The Civil Registration System was used to collect additional data on patient characteristics from Statistics Denmark (i.e. ethnicity, living status, urbanisation, income, and education level). The Danish National Health Service Register provided information on the number of contacts with OOH primary care. The National Patient Register provided data for calculating the Charlson Comorbidity Index, and the Register of Authorised Health Personnel provided information on the year of registration and specialization of the GPs.

### Data handling

We excluded telephone contacts resulting in referral to face-to-face consultation in OOH primary care, as these were seen as triage contacts to further care (without the option of an antibiotic prescription). In addition, we excluded GPs with less than two shifts on average per year for each type of contact. Therefore, a GP could be excluded from the analysis for one type of contact while contributing to the two other sets of analysis. ATC codes level 4 (chemical subgroup) and level 5 (chemical substance) were used to identify antibiotics, including J01 (antibacterial agents for systemic use).

We constructed a range of variables for case mix adjustment and stratifications (Supplementary Table 1). Two contact characteristics were defined: (1) ‘time to next in-hours period’, which measured the amount of time until daytime GP opening hours, and (2) ‘regional patient load, past hour’, which measured the overall workload at the entire regional OOH primary care service at the time of the contact. In addition, the patient characteristic ‘patient GP/OOH contacts in the past 12 months’ was constructed to describe the patients’ overall primary care utilization. For GP characteristics, we defined ‘OOH shifts in the past 180 days’ as a proxy of familiarity with working in OOH primary care and ‘patients seen in the past hour’ as a proxy for the activity of the individual GP at the time of the contact. We could not calculate ‘OOH shifts in the past 180 days’ if the contact took place within the first 180 days of the study period or ‘patients seen in the past hour’ if the contact took place during the first hour of the shift. These contacts were analysed as a separate category for completeness, but they were not presented in the final results. When calculating the complete workload for telephone shifts, we included telephone consultations and telephone triage contacts ending with a referral to a face-to-face consultation. If necessary, a missing category was introduced for each covariate.

### Analyses

To study GP variation in antibiotic prescribing (aim 1), we calculated the GPs’ unadjusted antibiotic prescribing rate, that is, the number of prescriptions per 100 contacts, and a prescribing tendency measure for each individual GP. This allowed us to adjust for case-mix variation between GPs, making the GP’s antibiotic prescribing tendency independent of his/her patient population. Therefore, we aggregated the data by covariate combinations and GP authorisation ID, and constructed a model for predicting prescription rates based on all before-mentioned patient- and contact-related characteristics using a Poisson regression model for each contact type separately [24]. This allowed us to estimate the number of expected antibiotic prescriptions for each GP given their particular case-mix. Second, we counted the number of observed prescriptions for each individual GP. Dividing the number of observed prescriptions by the number of expected prescriptions for each individual GP resulted in the antibiotic prescribing tendency (APT). The APT reflects the individual GP’s likelihood of prescribing antibiotics compared to the average GP. Thus, an APT >1 means that the GP is more likely to prescribe antibiotics than the average GP, and an APT <1 means that the GP is less likely to prescribe antibiotics than the average GP (adjusted for all before-mentioned patient- and contact-related characteristics). In other words, a GP with an APT of 1.2 prescribed antibiotics 20% more frequently than the average GP (adjusted for the above patient- and contact-related characteristics).

To obtain an objective measure of the APT variation among GPs, we sorted GPs according to APT and calculated the ratio between the 90th and 10th percentile. If a lot of variation is seen in APT among GPs, the distance between the 90th and 10th percentile will be wide, and the p90/p10 will be large. However, some variation among GPs will occur due to unobservable factors and random variation. To account for this, we estimated the amount of variation to be expected if all GPs acted exactly the same, that is if all GPs behaved as predicted by the Poisson model. For this, we used the estimated likelihood of antibiotic prescription for each consultation (from the Poisson model) as the mean in a binomial distribution and drew from that 100 random samples. This left us with 100 simulated datasets, which were each analysed in the same way as the real-world data, that is, for each of the 100 random samples, we calculated p90 and p10. Next, we calculated the median p90 and median p10 of these 100 random samples. Finally, we calculated the ratio between these median simulated p90 and p10, and compared this ratio to the observed p90/p10. The ratio between the observed p90/p10-ratio and the simulated p90/p10-ratio provided a measure of the excess variation in APT, that is, the amount of variation left after subtracting the expected variation from the observed variation. We refer to this as the expected variation, and it may be interpreted as the amount of observed variation that we would expect even if all GPs acted the same (i.e. if no true GP variation existed). We plotted these simulated APTs with the actual APTs alongside histograms of the unadjusted prescription rates. An alternative tool for studying excess variation, so-called funnel plots, were also constructed and showed similar results as the main analysis (Supplementary File).

To explore GP characteristics associated with antibiotic prescribing in OOH primary care (aim 2), we investigated GP-specific predictors of the APT by studying associations between GP and context characteristics and the observed APTs through the use of a multivariable Poisson model. Due to collinearity between GP age and experience, only age was included in the multivariable model. All regression models included cluster-robust variance estimation at the GP level to account for repeated measurements. All analyses were conducted in Stata 16 (StataCorp LP, College Station, TX, USA).

## Results

### OOH contacts, patient- and GP-related characteristics and antibiotic prescription rate

From 2014 to 2017, the OOH primary care service in the Central Denmark Region provided 794,220 clinic consultations, 281,141 home visits, and 1,583,919 telephone consultations (Tables 1 and 2). We excluded 39% of 954 GPs for clinic consultations (5.5% of contacts), 15% of 974 GPs for home visits (1.7% of visits), and 35% of 987 GPs for telephone consultations (2.0% of contacts). Table 2 presents the distribution of contact- and patient-related characteristics by contact type. The antibiotic prescription rate varied between contact types: 5.8% for telephone consultations, 11.6% for home visits, and 16.1% for clinic consultations. The range of quintiles for contact and GP characteristics is presented in Supplementary Table 2. GP characteristics on GP level can be found in Supplementary Table 3.

**Table 1.** Flow of data, per contact type (*n*).

	Clinic consultation				Home visit				Telephone consultation			
	Contacts	Patients	GPs	Prescriptions	Contacts	Patients	GPs	Prescriptions	Contacts	Patients	GPs	Prescriptions
	853,036	483,178	955	^a^	292,319	150,889	975	^a^	2,816,720	852,086	898	^a^
Merging of contacts and prescriptions	↓				↓				↓			
	853,036	483,178	955	137,160	292,319	150,889	975	33,657	2,816,720	852,086	898	94,258
Excluding contacts with invalid GP ID	↓				↓				↓			
	840,381	478,697	954	134,189	286,085	148,560	974	32,901	2,781,046	847,509	897	92,915
Exclusion due to too few contacts	↓				↓				↓			
	794,220	461,170	584	127,539	281,141	146,844	829	32,496	2,724,556	840,519	585	91,304
Removal of contacts ending in referral	↓				↓				↓			
	794,220	461,170	584	127,539	281,141	146,844	829	32,496	1,583,919	624,953	585	91,244

^a^Initial number of antibiotic prescriptions was 275,466 spread across 265,075 contacts with OOH primary care.

**Table 2.** Contact-, patient- and GP-related characteristics, proportion by contact type (in %).

	Clinic consultation		Home visit		Telephone consultation	
	N	%	N	%	N	%
Number of contacts	794,220	29.9	281,141	10.6	1,583,919	59.6
Antibiotic prescriptions	127,539	16.1	32,496	11.6	91,244	5.8
Contact characteristics						
Time to next in-hours period (hours)						
0–16	350,484	44.1	143,651	51.1	745,349	47.1
>16	443,736	55.9	137,490	48.9	838,570	52.9
Regional patient load, past hour						
First hour of shift	65,316	8.2	3263	1.2	136,866	8.6
1st quintile	150,601	19.0	60,708	21.6	292,980	18.5
2nd	146,371	18.4	55,138	19.6	296,210	18.7
3rd	148,032	18.6	59,323	21.1	280,398	17.7
4th	140,562	17.7	51,981	18.5	288,534	18.2
5th quintile	143,338	18.0	50,728	18.0	288,931	18.2
Patient characteristics						
Age in groups (years)						
0–3	123,626	15.6	11,142	4.0	237,778	15.0
4–17	163,130	20.5	13,032	4.6	231,450	14.6
18–39	253,280	31.9	39,198	13.9	499,454	31.5
40–64	183,704	23.1	66,315	23.6	351,052	22.2
≥65	70,480	8.9	151,454	53.9	264,185	16.7
Sex						
Female	409,657	51.6	147,611	52.5	893,919	56.4
Male	384,563	48.4	133,530	47.5	690,000	43.6
Highest educational level (years)						
<10	207,886	26.2	123,117	43.8	467,248	29.5
10–15	230,679	29.0	85,933	30.6	453,365	28.6
>15	86,627	10.9	26,187	9.3	188,087	11.9
Children	250,065	31.5	19,769	7.0	418,478	26.4
Missing values	18,963	2.4	26,135	9.3	56,741	3.6
Income^a^ (deciles)						
1st–3rd	249,452	31.4	109,575	39.0	542,473	34.2
4th–7th	329,701	41.5	129,774	46.2	675,526	42.6
8th–10th	215,067	27.1	41,792	14.9	365,920	23.1
Living status						
Married/cohabitating	543,295	68.4	127,988	45.5	954,064	60.2
Unmarried/widow(er)/divorced	250,925	31.6	153,153	54.5	629,855	39.8
Ethnicity						
Native born	699,824	88.1	262,125	93.2	1,425,728	90.0%
1st generation immigrants	57,638	7.3	15,458	5.5	97,344	6.1
2nd generation immigrants	36,758	4.6	3,558	1.3	60,847	3.8
Urbanisation (no. of inhabitants)						
>100,000	161,894	20.4	43,176	15.4	362,605	22.9
20,000–100,000	219,290	27.6	76,817	27.3	414,781	26.2
1000–20,000	214,882	27.1	98,585	35.1	456,862	28.8
<1000	174,718	22.0	58,792	20.9	306,327	19.3
Missing values	23,436	3.0	3771	1.3	43,344	2.7
Charlson Co-morbidity Index						
No comorbidities	681,020	85.7	140,675	50.0	1,253,337	79.1%
1	87,498	11.0	73,295	26.1	211,581	13.4
2	18,577	2.3	39,465	14.0	73,889	4.7
3	5072	0.6	17,584	6.3	28,376	1.8
≥4	2053	0.3	10,122	3.6	16,736	1.1
Patient GP/OOH contacts in the past 12 months (quintiles)						
1st quintile	196,013	24.7	62,579	22.3	385,635	24.3
2nd	170,531	21.5	52,170	18.6	266,394	16.8
3rd	123,772	15.6	54,829	19.5	316,520	20.0
4th	147,121	18.5	58,111	20.7	317,791	20.1
5th quintile	156,783	19.7	53,452	19.0	297,579	18.8
GP characteristics						
Number of GPs	584	29.2	829	41.5%	585	29.3
Sex						
Female	305,467	38.5	118,443	42.1	587,378	37.1
Male	488,753	61.5	162,698	57.9	996,541	62.9
Age in groups (years)						
31–40	154,484	19.5	59,987	21.3	277,527	17.5
41–50	303,572	38.2	103,873	36.9	548,966	34.7
51–60	221,466	27.9	75,254	26.8	497,531	31.4
>60	114,698	14.4	42,027	14.9	259,895	16.4
GP experience (years)						
6–10	118,123	14.9	49,537	17.6	204,233	12.9
11–20	346,430	43.6	115,259	41.0	633,477	40.0
>20	329,667	41.5	116,345	41.4	746,209	47.1
Primary care specialist						
No	132,270	16.7	46,339	16.5	256,698	16.2
Yes	404,080	50.9	141,992	50.5	698,033	44.1
Missings	257,870	32.5	92,810	33.0	629,188	39.7
OOH shifts in the past 180 days, quintiles						
First 180 days of follow-up	100,772	12.7%	37,160	13.2%	203,848	12.9%
1st quintile	159,487	20.1%	53,781	19.1%	286,159	18.1%
2nd	136,566	17.2%	48,390	17.2%	282,160	17.8%
3rd	124,639	15.7%	50,214	17.9%	272,232	17.2%
4th	141,608	17.8%	44,278	15.7%	271,064	17.1%
5th quintile	131,148	16.5%	47,318	16.8%	268,456	16.9%
Patients seen in the past hour, quintiles						
First hour of shift	129,878	16.4%	89,847	32.0%	246,562	15.6%
1st quintile	202,711	25.5%	106,350	37.8%	309,319	19.5%
2^nd^	106,885	13.5%	0	0.0%	275,376	17.4%
3^rd^	112,784	14.2%	68,429	24.3%	271,272	17.1%
4^th^	161,247	20.3%	0	0.0%	246,581	15.6%
** **5th quintile	80,715	10.2%	16,515	5.9%	234,809	14.8%

^a^We used parental characteristics to categorise children.

### GP variation in prescription rate and APT

**Figure 1.**
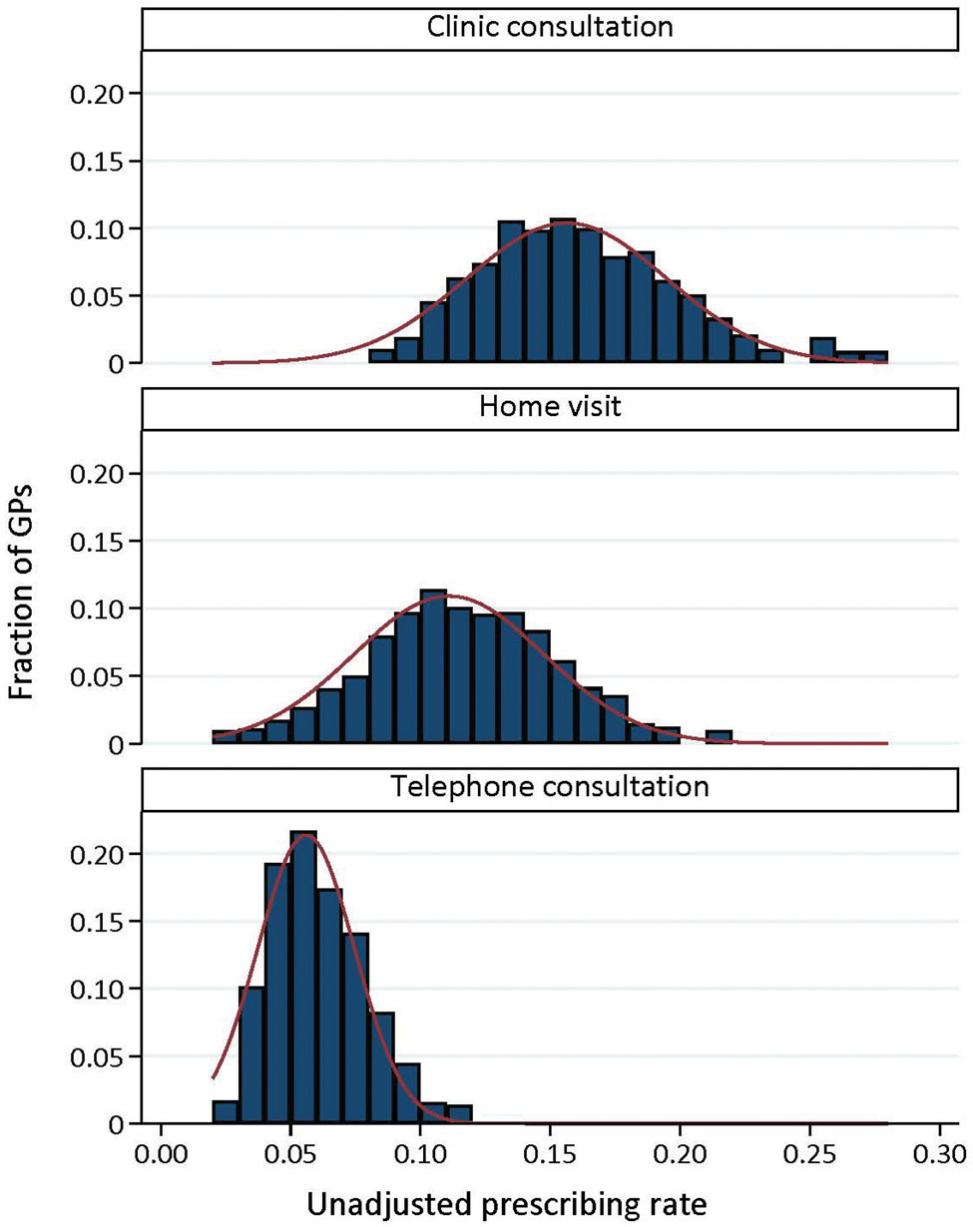
Histogram presenting raw antibiotic prescription rates, per contact type.

**Figure 2.**
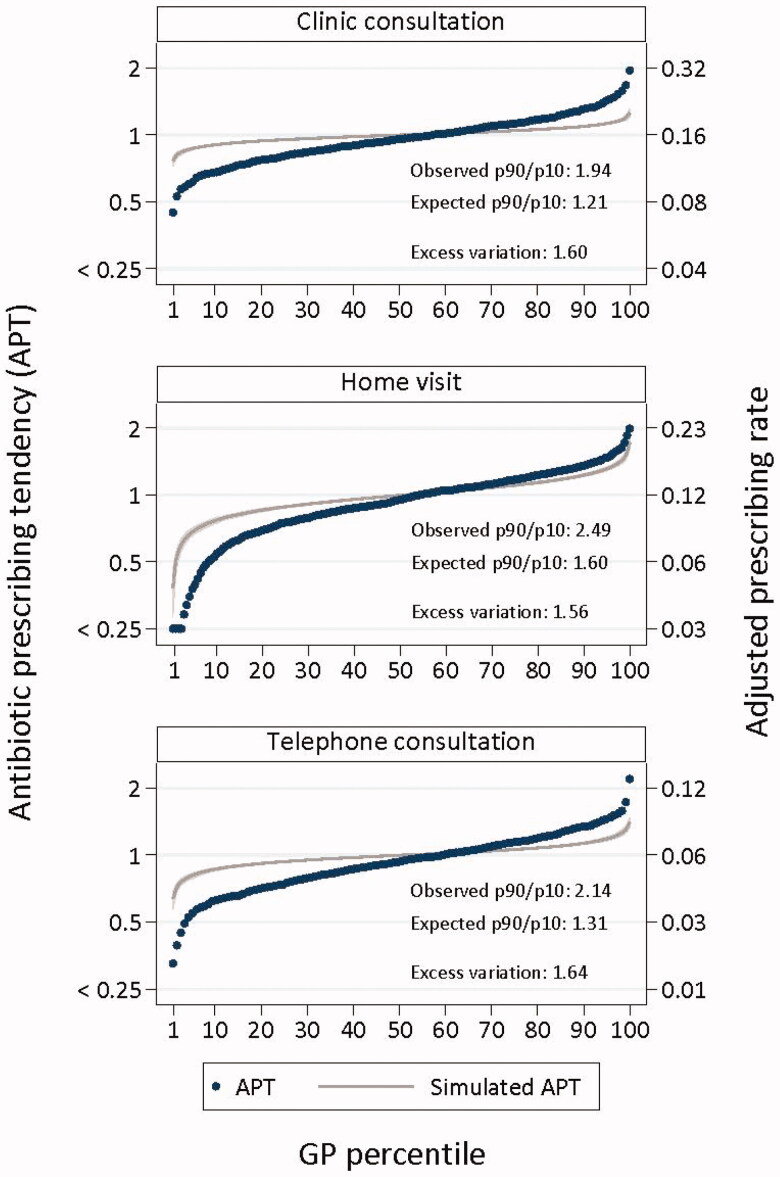
Adjusted^a^ APTs, per contact type. The simulated curves (grey) represent the situation in which all GPs act similarly. ^a^Adjusted for contact characteristics (year, month, time to next in-hours period, and patient load regionally past hour) and patient characteristics (age, sex, education level, income, living status, ethnicity, urbanisation, comorbidity, and patient GP/OOH contacts in the past 12 months). Left *Y*-axis: The adjusted APT presents the individual GP’s likelihood of prescribing antibiotics compared to the average GP. Right *Y*-axis: The APT is converted to an adjusted prescribing rate by multiplying the APT by the observed average prescribing rate for each contact type.

The histograms of the raw prescription rates show an approximately normal distribution of prescription rates for each contact type, with the range being the smallest for telephone consultations (Figure 1). The S-curves present the APTs adjusted for patient characteristics. The observed variation (p90/p10 ratio) was 1.90 for clinic consultations, 2.15 for telephone consultations, and 2.52 for home visits (Figure 2). The excess variation (i.e. not attributable to chance) in APT was 1.56 for clinic consultations, 1.64 for telephone consultations, and 1.58 for home visits.

### GP characteristics related with APTs

**Figure 3.**
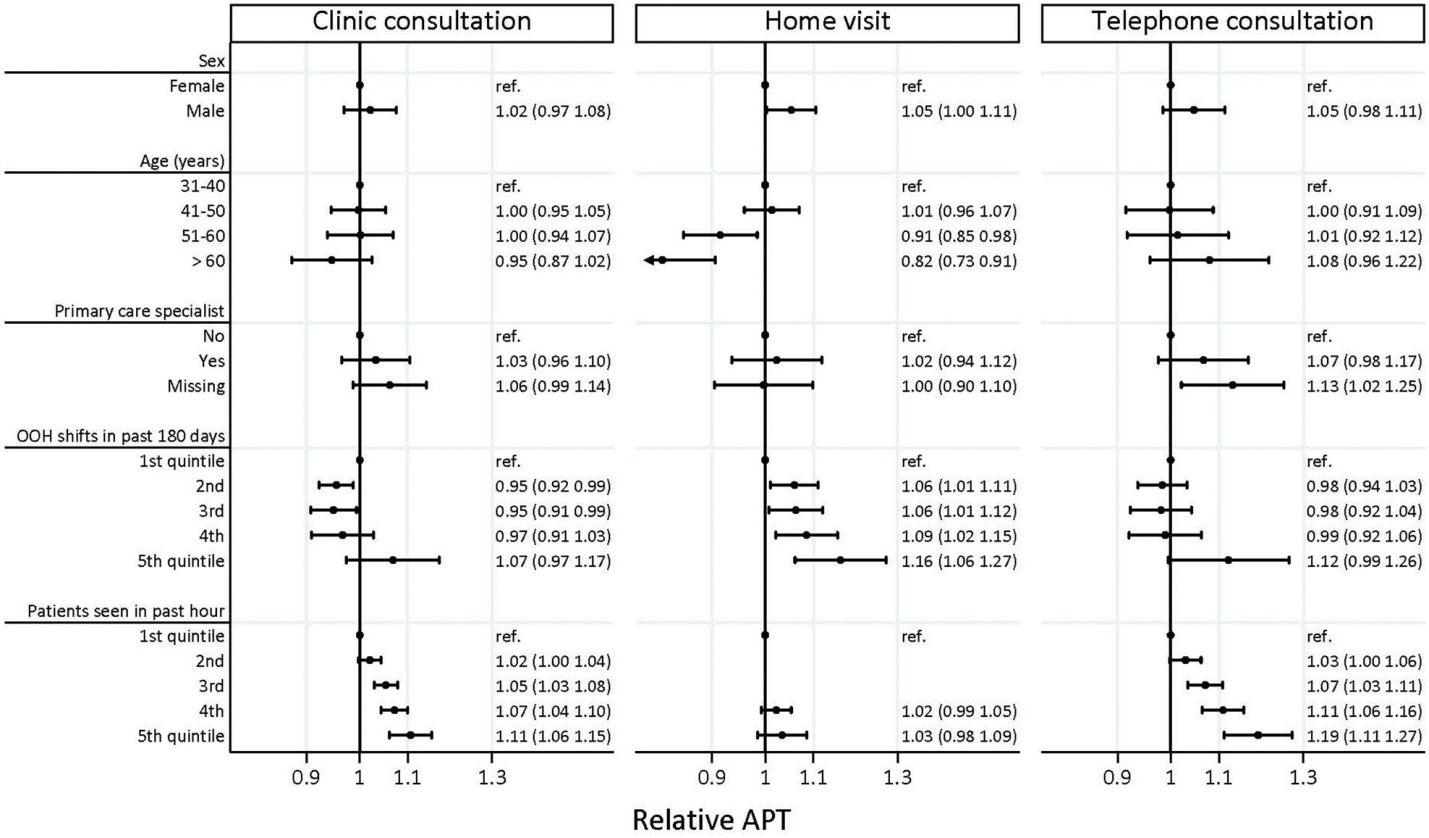
Adjusted relative risk of antibiotic prescribing tendency (APT) according to GP characteristics, stratified by contact type. Antibiotic prescribing tendency (APT) is the tendency of each individual GP to prescribe antibiotics compared to the average GP, calculated by dividing the number of observed antibiotic prescriptions by the number of expected prescriptions predicted by a model correcting for the case-mix of the patient population of each individual GP. Presented estimates were mutually adjusted, meaning, for example, GP sex effect was adjusted for GP age and vice versa. OOH shifts past 180 days refer to the number of similar shifts at out-of-hours (OOH) primary care done by the individual GP. Patients seen in the past hour refers to the total number of patients seen in the hour up to the index contact.

Figure 3 shows a forest plot presenting relative APTs for a range of GP characteristics, including 95% confidence intervals. For clinic consultations, familiarity with OOH care and activity level were significantly correlated with relative APT; high familiarity with OOH care was correlated with a lower relative APT (compared to the lowest level of familiarity), and high activity level was correlated with a higher relative APT (compared to the lowest level of activity). For home visits, sex, age, and familiarity with OOH care were significantly correlated with relative APT; males had higher relative APT than women, older GPs had lower relative APT than young GPs, and high familiarity with OOH care was correlated with a higher relative APT (compared to the lowest level of familiarity). The number of patients seen in the past hour was positively correlated with relative APT for telephone consultations. Generally, the GP characteristics explained very little of the variation, with most estimates being close to 1 and statistically insignificant. In our regression model, the GP characteristics explained between 0.8% and 2.5% of the variation in APT (Pseudo *r*^2^: 0.010 for clinical consultations, 0.008 for home visits, and 0.025 for telephone consultations).

## Discussion

### Statement of principal findings

GPs have varying antibiotic prescribing rates in OOH primary care in Denmark; this was seen for each type of investigated contact. The variation is 56–64% larger than would be expected if every GP acted the same. The included GP characteristics explained little of the variation among GPs, but some were significantly related to higher relative APT; activity level of the individual GP (for clinic consultations and home visits), familiarity with working at the OOH primary care, male sex, and younger age (all for home visits). Average familiarity with working at the OOH primary care correlated with lower antibiotic prescribing rates for clinic consultations.

### Strengths and weaknesses of the study

The large dataset with unique data on individual GPs enabled us to study GP variation in antibiotic prescribing in OOH primary care. We developed our own measure of excess variation, adjusting for patient- and contact-related characteristics and taking into account that some variation will exist even when GPs act the same. The use of retrospective routine care data implied that the GPs were not aware of the analyses of their prescribing behaviour, which removed the risk of bias from socially desirable prescribing behaviour. Furthermore, the linking of data across national registers allowed us to adjust for many patient- and contact-related characteristics, thereby limiting confounding.

The quality of our data is unknown, but we assume that the data has high validity. We used billing data from GPs, who are paid on a fee-for-service basis in OOH primary care. In addition, antibiotic prescriptions are made directly in the OOH primary care registration system, which limits the risk of incorrect prescription data. Data on prescriptions and contacts came in two essentially independent datasets, which we merged by the personal identification number and timestamp. Few prescriptions (3.8%) could not be linked to a contact and were excluded. As we collected the data directly from the OOH primary care system, we obtained data from only one of the five Danish regions, which could have influenced the generalisability. However, since the organisation of the OOH primary care services in the Central Denmark Region is largely similar to the organisation in the other three regions served by a GP cooperative, we expect the results to be representative for Danish OOH primary care. Only the Capital Region of Denmark has a different organisation (i.e. medical helpline 1813) without clinic consultations by GPs. Furthermore, the results can be generalised to settings with similar OOH primary care services in other countries with low antibiotic prescribing rates. We assume that GP characteristics associated with APT are largely similar to those in other countries with a GP gatekeeping system. Only limited information was available on GP characteristics in the Danish registers, and GP characteristics explained only little of the variation seen. Consequently, a range of other characteristics could be relevant, such as communication skills and personality characteristics [10]. We cannot rule out additional confounding from unobserved covariates or residual confounding. Finally, our contact data lacked information on diagnoses and reason for the encounter. This information could have improved the precision of the prediction model. However, in consideration of the vast amount of data, this is unlikely to have influenced our findings.

### Findings in relation to other studies

We are not aware of other studies that have investigated GP variation in antibiotic prescribing in OOH primary care. However, many studies have investigated variation in daytime GP practices, mostly at the practice level, and have reported great variation in antibiotic prescribing rates between diagnoses and practices [6,7,9]. Besides different settings, comparison of these studies is difficult due to differences in the measures used. The percentage of contacts ending with an antibiotic prescription was commonly used while correcting for case mix and/or standardising between practices. By calculating excess variation, we took into account that some variation would be observed between GPs, even if no true variation was present (if all GPs acted exactly the same). This method described variation more accurately, but it complicated comparison with existing literature that did not take this random variation into account.

In the daytime, GP activity level (i.e. practice activity, consultation rates, feeling of busyness) was associated with antibiotic prescriptions [25,26]. In line with other studies in OOH primary care, we found that GP activity level was related to antibiotic prescribing. Lindberg et al. found that antibiotic prescribing for acute respiratory tract infections increased during busy sessions [5]. In a qualitative study by Williams et al., the GPs indicated that prescribing antibiotics is sometimes easier than starting a discussion with a patient during a busy OOH shift [18].

### Meaning of the study

The GPs in OOH primary care had varying antibiotic prescribing rates, which suggests room for improvement, but further research is needed to move towards clinical recommendations. In our study, the activity level of the individual GP and the GP’s familiarity with working at the OOH primary care had the strongest associations with antibiotic prescribing among the investigated GP characteristics. Activity can be seen as a GP-related and/or context-related characteristic. On the one hand, the GPs can influence the number of patients they handle, for example by speeding up and taking fewer or shorter breaks. On the other hand, the number of patients seen per hour is, at least partly, an indicator of the overall busyness in the clinic; if the patient load is high, a GP could see more patients per hour. When many patients are seen (high activity), the GPs may feel the pressure of time constraints; the GP may not be able to discuss alternative approaches or the nature of the illness with the patient, and the GP may ultimately prescribe antibiotics as a time-saving strategy [27]. The GPs could also be less motivated to discuss the necessity of an antibiotic prescription with a patient during busy OOH shifts [20] as they do not see their own patients, for whom such time investment may seem more relevant. Furthermore, the fee-for-service payment might affect the activity level because the GPs get paid more when handling more patients, which holds an incentive to work faster.

As the GP characteristics included in our study explained only a limited part of GP variation, future studies could focus on identifying other relevant characteristics that may affect the OOH antibiotics prescribing behaviour of GPs, such as interest in antibiotic prescribing, emotional state [22], personal habits [28], ideas about overuse of antibiotics [26], limited access to patient records [20], and the clinician’s perception that a patient expected an antibiotic prescription [10]. It is also relevant to consider the GPs’ antibiotic prescribing rate in the daytime. Furthermore, organisational characteristics might explain some of the variation. Access to and use of POC tests could be a relevant factor [7]. These tests have been introduced to support the GPs’ decision-making to reduce the number of unnecessary antibiotic prescriptions [29].

Studying variation in prescribing helps to pinpoint both low and high antibiotic prescribers, which can be used to tailor interventions [30]. We could learn from the low prescribers when developing interventions that focus on the large middle group and from high prescribers when aiming to reduce the antibiotics prescribing rate across all GPs. Qualitative research is warranted to get more insight into the decision-making process and to explore the considerations of low prescribers. Identified modifiable factors could be operationalised and measured quantitatively to estimate their relative importance in explaining variation. Finally, possible negative consequences of reducing antibiotic prescribing should be considered. When aiming at reducing antibiotic prescriptions where possible (increasing specificity), one might miss patients who actually need antibiotics (decreasing sensitivity). Both errors come at different costs, and the cut-off point for accepting the risk of an error (and subsequent costs) may be different for GPs in OOH care than for GPs in daytime care.

## Ethical approval

We received approval from the Danish Patient Safety Authority (no. 3-3013-2692/1) for using data from the regional OOH primary care system. Furthermore, the project is listed in the record of processing activities at the Research Unit for General Practice in Aarhus in accordance with the provisions of the General Data Protection Regulation (GDPR).

## Supplementary Material

Supplemental Material
