# Supplementary material for: Variation of GP antibiotic prescribing tendency for contacts with out-of-hours primary care in Denmark – a cross-sectional register-based study
Source: Scand J Prim Health Care. 2022 Jun 15;40(2):227–36. doi: 10.1080/02813432.2022.2073981 (PMC9397449; doi:10.1080/02813432.2022.2073981)
Supplement: Supplemental Material [file IPRI_A_2073981_SM9846.docx]

**Supplementary table 1.** Covariate list contact-, patient-, and GP-related characteristics

| **Covariate** | **Type** | | **Categories** | **Label** | **Origin** |
| --- | --- | --- | --- | --- | --- |
| ***Contact characteristics*** | | | | | |
| **Year** | Categorical | | Years, range 2014-2017 | Year of contact | Contact data |
| **Month** | Categorical | | Months, range January-December | Month of contact | Contact data |
| **Time to next in-hours period** | Binary | | 0-16 hours, >16 hours | Amount of time until daytime GP opening hour | Estimated from contact data |
| **Regional patient load, past hour** | Categorical (quintiles) | | Quintiles by contact, with a separate category for the first hour of a shift | Overall workload at the entire regional OOH primary care service at the time of the contact | Estimated from contact data |
| ***Patient characteristics*** | | | | | |
| **Age** | Categorical for tables and figures, four knotted spline for estimating APT | | Five categories (in years):0-3, 4-17, 18-39, 40-64, ≥65 | Patient’s age | Contact data |
| **Sex** | Binary | | Female, male | Patient’s sex | Contact data |
| **Highest educational level** | Categorical | | Five categories: <10 years, 10-15 years, >15 years, children, unknown | Total years of education for patient | Statistics Denmark |
| **Income** | Categorical | | Income deciles grouped into three categories (in deciles): 1^st^-3^rd^, 4^th^-7^th^, 8^th^-10^th^ | Disposable income, adjusted for size of household | Statistics Denmark |
| **Living status** | Binary | | Married/cohabiting, unmarried/widow(er)/divorced | Patient’s living status | Statistics Denmark |
| **Ethnicity** | Categorical | | Native born, 1^st^ generation immigrant, 2^nd^ generation immigrant | Patient’s ethnicity | Statistics Denmark |
| **Urbanisation** | Categorical | | Four categories according to size: >100,000, 20,000-100,000, 1,000-20,000, <1,000, not placeable | Number of people within patient’s city | Statistics Denmark |
| **Charlson Co-morbidity Index** | Categorical | | Number of comorbidities as defined by Charlson: No comorbidities, 1, 2, 3, ≥4 | Patient’s co-morbidity | Calculated from the National Patient Registry |
| **Patient GP/OOH contacts in the past 12 months** | Categorical | | Quintiles by contact | Patient’s overall primary care utilization | Calculated from National Health Service Register |
| ***GP characteristics*** | | | | | |
| **Sex** | | Binary | Female, male | GP’s sex | Authorisation register |
| **Age** | | Categorical | Four categories (in years): 31-40, 41-50, 51-60, >60 | GP’s age | Authorisation register |
| **GP experience** | | Categorical | Three categories (in years): 6-10, 11-20, >20 | Years since finishing MD | Authorisation register |
| **Primary care specialty** | | Binary | Yes, no, missing values | Registration as primary care specialist | Authorisation register |
| **OOH shifts in the past 180 days** | | Categorical | Quintiles by contact, with separate category for the first 180 days of the study period | Proxy for familiarity with working at the OOH primary care | Estimated from contact data |
| **Patients seen in the past hour** | | Categorical | Quintiles by contact, with separate category for the first hour of a shift | Proxy for activity of the individual GP at the time of the contact | Calculated from contact data |

**Supplementary file.** Funnel plots

*Methods*

Funnel plots as introduced by Light and Pillemer [1], and repurposed for contexts similar to this by Spiegelhalter [2], are useful for detecting outliers on a given performance measure. One such performance measure is the APT. By plotting APT as a function of the expected number of antibiotic prescriptions, one may get a sense for excess variation, by counting the number of GPs who deviate significantly from the expectation of the model.

We constructed the funnel plots with the FunnelPlotR package for R, using the APT model to generate predictions. GPs who showed prescription rates outside 95% and 99.8% confidence intervals were considered outliers. Results were adjusted for overdispersion by log-transformation and truncation of the distribution, as described in the FunnelPlotR documentation [3]. Due to the strict anonymity restrictions of Statistics Denmark, we are not allowed to show the plots. Therefore, we presented aggregated results (Table).

**Table.** Number of GPs per group of predicted prescriptions and percentage of GPs being outliers (95% and 99.8%)

| **Contact type** | **Predicted prescriptions** | **GPs**  **(N)** | **Outliers 95% level**  **N (%)** | **Outliers 99.8% level**  **N (%)** |
| --- | --- | --- | --- | --- |
| **Clinic consultation** | <150 | 280 | 48 (17%) | 10 (4%) |
|  | 150-300 | 191 | 31 (16%) | 8 (4%) |
|  | >300 | 113 | 29 (26%) | 10 (9%) |
|  | Total | 584 | 108 (18%) | 28 (5%) |
| **Home visit** | <20 | 248 | 46 (19%) | 18 (7%) |
|  | 20-60 | 431 | 66 (15%) | 23 (5%) |
|  | >60 | 150 | 35 (23%) | 16 (11%) |
|  | Total | 829 | 147 (18%) | 57 (7%) |
| **Telephone consultation** | <100 | 311 | 57 (18%) | 11 (4%) |
|  | 100-250 | 180 | 40 (22%) | 10 (6%) |
|  | >250 | 94 | 19 (20%) | 6 (6%) |
|  | Total | 585 | 116 (20%) | 27 (5%) |

One would expect about two in 1,000 GPs to end up outside the 99.8% interval by chance alone. As we find figures in the range of 50-70 per 1,000 across all contact types, this method also clearly illustrated that our dataset has a lot of excess variation.

*References*

1. Light RJ, Pillemer DB. Summing up. The science of reviewing research. Cambridge:Harvard University Press;1984.
2. Spiegelhalter DJ. Funnel plots for comparing institutional performance. Stat Med. 2005;24:1185-202.
3. The Comprehensive R Archive Network. Package ‘FunnelPlotR’. September, 2021. Available from: <https://cran.r-project.org/web/packages/FunnelPlotR/FunnelPlotR.pdf>

**Supplementary table 2.** Overview of range per quintile for selection of variables, per type of contact (n)

|  | **Clinic consultations** | | **Home visits** | | **Telephone consultations** | |
| --- | --- | --- | --- | --- | --- | --- |
| **Variable,** quintiles | **Min** | **Max** | **Min** | **Max** | **Min** | **Max** |
| **Regional patient load, past hour** |  |  |  |  |  |  |
| - 1^st^ | 0 | 47 | 0 | 8 | 0 | 96 |
| - 2^nd^ | 48 | 57 | 9 | 11 | 97 | 153 |
| - 3^rd^ | 58 | 66 | 12 | 14 | 154 | 180 |
| - 4^th^ | 67 | 76 | 15 | 17 | 181 | 220 |
| - 5^th^ | 77 | 143 | 18 | 34 | 221 | 445 |
| **Patient GP/OOH contacts in the past 12 months** |  |  |  |  |  |  |
| - 1^st^ | 0 | 2 | 0 | 6 | 0 | 3 |
| - 2^nd^ | 3 | 5 | 7 | 12 | 4 | 6 |
| - 3^rd^ | 6 | 8 | 13 | 20 | 7 | 11 |
| - 4^th^ | 9 | 14 | 21 | 34 | 12 | 21 |
| - 5^th^ | 15 | 300 | 35 | 300 | 22 | 300 |
| **OOH shifts in the past 180 days** |  |  |  |  |  |  |
| - 1^st^ | 1 | 13 | 1 | 9 | 1 | 13 |
| - 2^nd^ | 14 | 18 | 10 | 14 | 14 | 19 |
| - 3^rd^ | 19 | 24 | 15 | 20 | 20 | 26 |
| - 4^th^ | 25 | 35 | 21 | 30 | 27 | 36 |
| - 5^th^ | 36 | 85 | 31 | 85 | 37 | 85 |
| **Patients seen in the past hour** |  |  |  |  |  |  |
| - 1^st^ | 0 | 3 | 0 | 1 | 0 | 10 |
| - 2^nd^ | 4 | 5 |  |  | 11 | 14 |
| - 3^rd^ | 6 | 6 |  |  | 15 | 17 |
| - 4^th^ | 7 | 8 | 2 | 2 | 18 | 21 |
| - 5^th^ | 9 | 27 | 3 | 10 | 22 | 68 |

**Supplementary table 3.** GP characteristics on GP level

|  | **Clinic consultation** | | **Home visit** | | **Telephone consultation** | |
| --- | --- | --- | --- | --- | --- | --- |
|  | **N** | **%** | **N** | **%** | **N** | **%** |
| **N** | 584 | 29.2 | 829 | 41.5 | 585 | 29.3 |
| **Sex** |  |  |  |  |  |  |
| - Female | 277 | 47.4 | 440 | 53.1 | 279 | 47.7 |
| - Male | 307 | 52.6 | 389 | 46.9 | 306 | 52.3 |
| **Age (years)** |  |  |  |  |  |  |
| - 31-40 | 202 | 34.6 | 293 | 35.3 | 191 | 32.6 |
| - 41-50 | 189 | 32.4 | 267 | 32.2 | 188 | 32.1 |
| - 51-60 | 139 | 23.8 | 204 | 24.6 | 153 | 26.2 |
| - >60 | 54 | 9.2 | 65 | 7.8 | 53 | 9.1 |
| **GP experience (years)** |  |  |  |  |  |  |
| - 6-10 | 190 | 32.5 | 266 | 32.1 | 175 | 29.9 |
| - 11-20 | 195 | 33.4 | 282 | 34.0 | 198 | 33.8 |
| - >20 | 199 | 34.1 | 281 | 33.9 | 212 | 36.2 |
| **Primary care specialist** |  |  |  |  |  |  |
| - No | 145 | 24.8 | 188 | 22.7 | 138 | 23.6 |
| - Yes | 255 | 43.7 | 354 | 42.7 | 236 | 40.3 |
| - Missing values | 184 | 31.5 | 287 | 34.6 | 211 | 36.1 |
